# Fish Hook Injury: Removal by ‘’Push Through and Cut Off‘’ Technique: A Case Report and Brief Literature Review

**DOI:** 10.5812/traumamon.17728

**Published:** 2014-03-24

**Authors:** Hayat Ahmad Khan, Younis Kamal, Ansar ul Haq Lone

**Affiliations:** 1 Department of Orthopaedics, Bone and Joint Hospital, Kashmir, India

**Keywords:** Wounds and Injuries, Fish Hook, Hand Injuries

## Abstract

**Introduction:**

Fishing is a leisure activity for some people around the world. Accidently the fish hook can get hooked in the hand. If the hook is barbed, removal becomes difficult. We report a case of such a injury in the hand and discuss the technique for its removal with a brief review of the literature.

**Case Presentation:**

A thirty-two year old male accidently suffered a fishhook injury to his hand. He came to the orthopaedic ward two hours after the incident with pain; the fish hook was hanging from the hand. Unsuccessful attempts to remove it were made by his relatives. A push-through and cut-off technique was used for removal of barbed hook.

**Discussion:**

Barbed hooks are to be removed atraumatically with controlled incision over properly anaesthetised skin. Proper wound management and prophylactic antibiotics suitable for treatment of *Aeromonas* species should be initiated to prevent complications.

## 1. Introduction

Fishing is a leisure activity for many across the globe. Barbed hooks are used for more effectiveness . The front end of the hook is barbed so that it gets caught into the fish’s mouth. Accidently, it may snag the fisherman and cause hand or bodily injury. Most commonly it involves the hand or head . The external injury seems minimal but due to barbed hook, the internal injuries can be dangerous particularly when it is near to a vessel, tendon or nerve. Patients are usually accompanied by their relatives or friends who try to remove it blindly and often cause more damage to the soft tissues and do not provide proper wound care. 

## 2. Case Presentation

**Figure 1.**
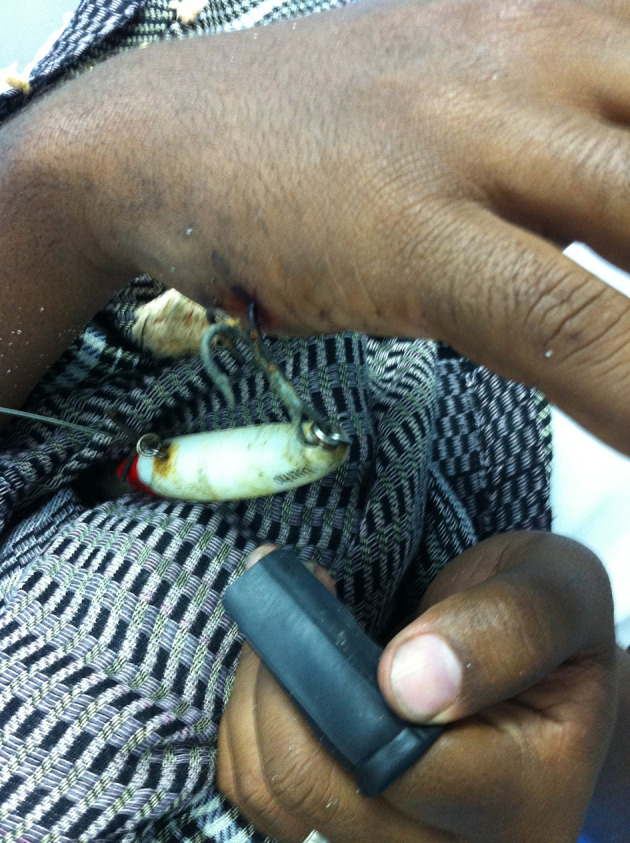
Fish Hook Hanging From the Hand

A 32 year-old male accidently sufferred a fishhook into his hand while fishing. He came to the orthopedic ward two hours after the incident with pain in and a fish hook hanging from his hand (Figure 1). Bleeding from the entry site was mild. Unsuccessful attempts at removal were made by his relatives. We prepared the patient for removal under local anaesthesia. Proper asepsis was ensured; then local anesthesia was administered using 1% lignocaine. The tip of the hook was palpated and advanced forwards. A small incision was given to make way for the barbed end. The end was located and held with small artery forceps. Since the hook was embedded deep into the muscle, it was cut by using a cutter below the barbed end. The remaining portion of the hook was backed-out via the entry site. Thorough cleaning of wound with normal saline and povidone iodine was performed. The patient was discharged with advice for follow-up .

**Figure 2.**
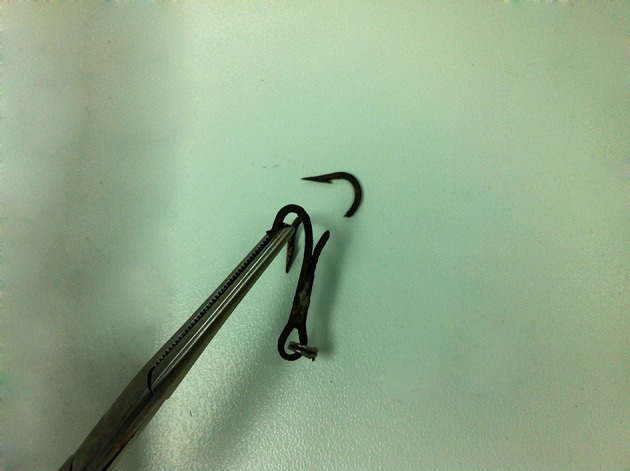
Removed Fish Hook

## 3. Discussion

Like many outdoor pursuits, fishing can, at times, be a dangerous pastime. This is not surprising when the most important piece of the fishing gear is a sharp curved metal hook. The potential dangers of a fish hook have been highlighted recently (1). The hand is most commonly injured followed by the head and eyes. Although the former injury can be managed in the emergency department, the latter needs specialized care (2). If one is not familiar with fishing gear, injury becomes more probable. With more modifications in the hook design, the emphasis is mostly on the barbed end. It is designed to snag the fish’s mouth and makes hook removal difficult. 

**Figure 3.**
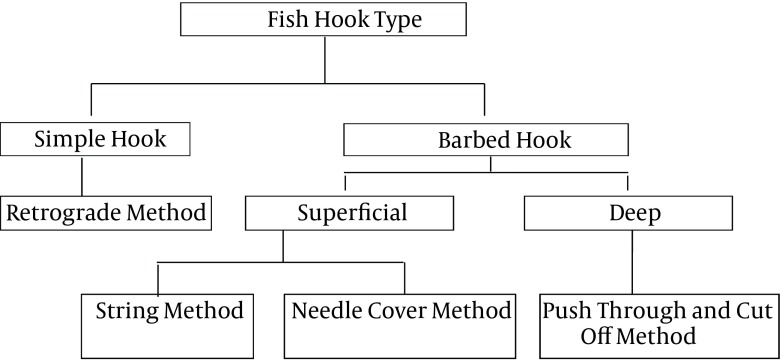
Fish Hook Removal Method Based on the Type of Hook and Penetration into Skin

**Figure 4.**
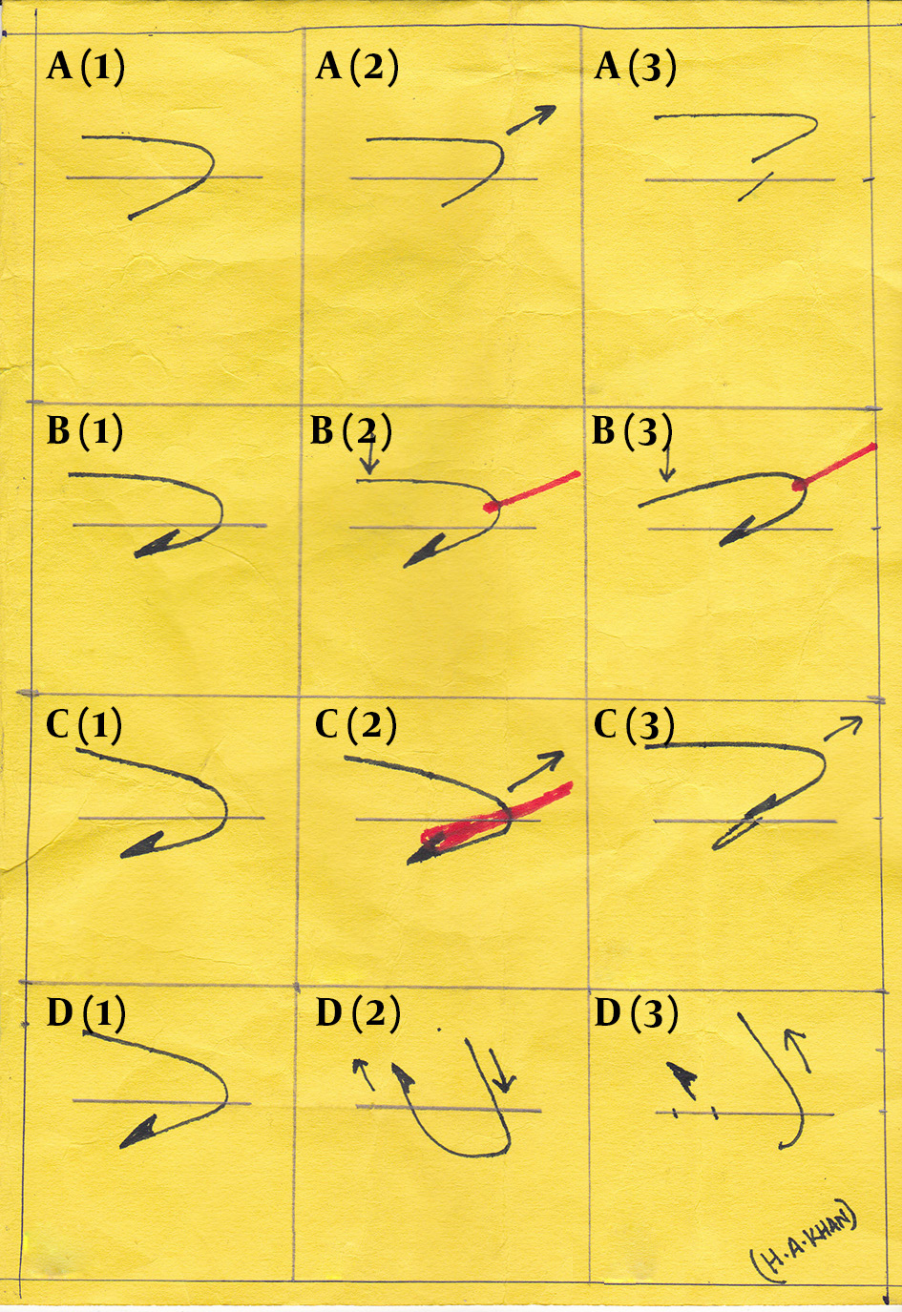
Technique of Fish Hook Removal A1-A3: Removal of simple hook by retrograde technique; B1-B3: Removal of Barbed hook by string pull method, Red line depicts the pull of thread and black arrow the downward force; C1-C3 Needle cover technique, red line indicates the needle covering the barb and black arrow shows the direction of force; D1-D3 Advance and cut off technique: Note the barbed end is pushed through a different site and the remaining shaft is backed out via the entry site.

No guideline is presently available in the literature for safe removal of fishhook to the best of our knowledge. Proposed techniques are as follows (Figures 3 and 4);

1. Retrograde technique
2. String pull technique
3. Needle cover technique
4. Advance and cut technique

Doser et al. (3) in a study on 100 patients found the retrograde technique useful in only 40 patients.

The string pull technique is the modified retrograde technique with the advantage of being less traumatic to soft tissues, and does not need another exit site. Cooke (4) described how to remove fish hook with a string which has undergone lot of modifications till now. However, its effectiveness on deeply embedded barbed hooks is questionable. Furthermore, in areas where the risk of damage to vascular structures is greater, this technique cannot be applied.

The needle cover technique can be effectively applied for superficially embedded barbed fishhooks. Being a blind procedure, its use for deeply embedded hooks in high risk neurovascular areas cannot be guaranteed. 

Prats et al. (2013) emphasized the management of injuries caused by barbed hooks but showed his method on only two cases (5). Nabi et al. discouraged home removal of fish hooks by unqualified persons in a single case report (6). However in their technique the barb was not cut as it was easily disengaged by slight rotation which was possible in the first web space of the hand. Two important aspects of our case were the neurovascular risk of deep branches of the ulnar nerve and artery and continuation of the ulnar bursa to the wrist and distal forearm respectively.

Several attempts of removal had been made by his relatives causing more soft tissue trauma. Further hypothenar muscles including the abductor digiti minimi, flexor digiti minimi brevis and opponens digiti minimi were all at risk of injury which is important for the normal functioning of hand especially gripping in the dominant right hand. The close association of ulnar nerve in the Guyon’s canal also makes the removal risky. (The deep branch of the ulnar nerve passes between the abductor digiti minimi and flexor digiti minimi).

The advance and cut method is used successfully in almost all types of barbed hooks (7) (as in our case). A small incision over the anaesthetised skin exposes the barbed tip. The hook is pushed through and the barbed end is cut under direct vision, thus minimizing injury to soft tissue or the underlying structures. The remaining part is backed out via the entry site. Whatever technique is used, wound care is of utmost importance. Thorough washing with aseptics and proper tetanus prophylaxis for unimmunized patients are the basic requirements of wound care. Though use of systemic antibiotics is not recommended for superficial wounds (3), prophylactic oral fluoroquinolones to cover *Aeromonas* *hydrophila* are recommended for deep wounds (8-10). Patients should be followed for proper healing and absence of infection. 

Skiendzielewski et al. (11) in one case report of wound infection due to fresh water contamination by *Aeromonas hydrophila* concluded that this pathogen must be suspected in all wounds occurring in fresh water. Semel et al. (12) in their study warned regarding rapidly progressive nature of soft tissue infections in water-associated traumatic wounds; 39% of their cases had associated fascia, muscle, tendon, bone or joint infections. Since the hook in our case was in close proximity to the ulnar bursa, thus, the chance of spread of infection to the forearm was high due to continuity of the bursa to the distal forearm. Thorough lavage, atraumatic removal and prophylactic antibiotics decrease the risk of infection.

### 3.1. Conclusions

Barbed hooks may be removed atraumatically with controlled incision over properly anaesthetised skin. Proper wound management and prophylactic antibiotics suitable for treatment of *Aeromonas* species should be considered to prevent complications. More investigations should be performed to assess the results of different techniques used for the removal of barbed fish hooks and the rate of infections caused by these injuries.
